# LIMK1 variants are associated with divergent endocrinological phenotypes and altered exocytosis dynamics

**DOI:** 10.1016/j.isci.2025.112585

**Published:** 2025-05-05

**Authors:** Irena J.J. Muffels, Theodore Carter, Holger Rehmann, Sebastiaan J. Vastert, Annemarie A. Verrijn Stuart, Andreas C. Blank, Aurore Garde, Bert van der Zwaag, Iris M. De Lange, Jacques C. Giltay, Koen L.I. van Gassen, Klaas Koop, Cedric S. Asensio, Peter M. van Hasselt

**Affiliations:** 1 Department of Metabolic Diseases, Wilhelmina Children’s Hospital, University Medical Center Utrecht, Utrecht, the Netherlands; 2 Department of Biological Sciences, College of Natural Sciences and Mathematics, Denver, CO, USA; 3 Department of Energy and Biotechnology, Flensburg University of Applied Sciences, Flensburg, Germany; 4 Department of Pediatric Rheumatology and Immunology and Center for Translational Immunology, Wilhelmina Children’s Hospital, University Medical Center Utrecht, Utrecht, the Netherlands; 5 Department of Pediatric Endocrinology, Wilhelmina Children’s Hospital, University Medical Center Utrecht, Utrecht, the Netherlands; 6 Department of Pediatric Cardiology, Wilhelmina Children’s Hospital, University Medical Center Utrecht, Utrecht, the Netherlands; 7 Centre de Génétique et Centre de Référence Maladies Rares, Fédération Hospitalo-Universitaire Médecine Translationnelle et Anomalies du Développement (FHU TRANSLAD), Hôpital d'Enfants, Centre Hospitalier Universitaire de Dijon, Dijon, France; 8 Department of Medical Genetics, Wilhelmina Children’s Hospital, University Medical Center Utrecht, Utrecht, the Netherlands

**Keywords:** Endocrinology, Biological sciences, Functional aspects of cell biology

## Abstract

LIM kinase 1 (LIMK1) plays a pivotal role in dynamic actin remodeling through phosphorylation of cofilin, thereby regulating exocytosis. We report two individuals harboring *LIMK1 de novo* variants with dissimilar phenotypes: one exhibited epileptic encephalopathy and developmental delay, while the other showed common variable immune deficiency and glucose dysregulation. We suspected that the divergent phenotypic features arose from opposing effects on LIMK1 activity. Indeed, actin polymerization was significantly decreased in individual 1, whereas it was increased in individual 2. Insulin-secreting cell lines expressing the *LIMK1* variant of individual 1 exhibited significantly slower exocytosis, contrasting the rapid and uncontrolled exocytosis in individual 2. Intriguingly, both variants led to increased overall insulin secretion. This first report of two individuals with *LIMK1* variants with divergent effects on cofilin phosphorylation and actin polymerization, reveals that LIMK1 has an important role in tuned insulin exocytosis. These distinct exocytosis defects may underlie the glucose dysregulation observed.

## Highlights

• We found two disease-causing *LIMK1* missense variants in patients with divergent symptoms
• One patient shows seizures while the other shows immune- and glucose dysregulation
• The A43T variant boosts actin polymerization, while G511A stimulates depolymerization
• These opposing changes align with exocytosis dynamics and divergent clinical phenotypes

## Graphical abstract

**Figure:**
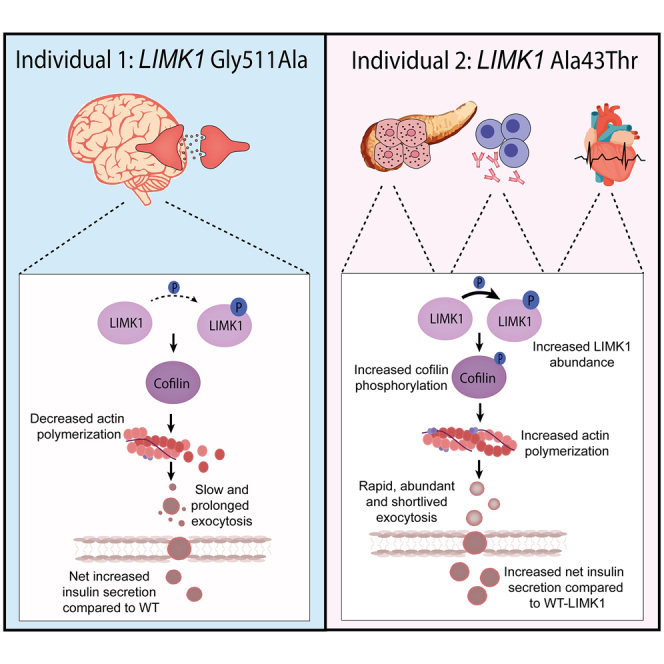

## Introduction

The unique ability of actin to provide structural support to cells, while allowing dynamic adaptations, is essential for eukaryotic health. The cytoskeletal ability to change rapidly is utilized in many ways, including cellular migration, endocytosis, and exocytosis.^1^^,^^2^ A set of actin-modifying proteins warrant appropriate tuning of the actin cytoskeleton when needed. The importance of these proteins is underlined by a growing number of monogenetic disorders caused by dysfunction of these proteins, called actinopathies.^3^

LIM kinase 1 (LIMK1) is an example of an actin modifying protein that operates via phosphorylation of cofilin, which increases the elasticity of the actin cytoskeleton by severing actin filaments.^4^ LIMK1 and cofilin operate in a complex signaling cascade, thought to allow rapid actin filament disassembly when needed.^5^ LIMK1 is activated by phosphorylation of residue 508, by the small GTPase Rho and its downstream protein kinase Rho-associated coiled-coil kinase (ROCK).^6^ In addition, the two LIM domains and the PDZ domain directly mediate LIMK1 activity, which is thought to occur through interaction with its own kinase domain.^7^^,^^8^ LIMK1 can also transphosphorylate other LIMK1 proteins, thereby increasing their activity, half-life, and stability.^9^ It is thought that these complex feedback dynamics promote unique protein kinetics, essential to facilitate cellular processes requiring rapidly changing morphology, such as endo- and exocytosis. However, the exact role of actin in these processes is still debated, as it can be either a positive or negative regulator, depending on the secretory system under investigation.^10^ For example, both disrupting and stimulating actin polymerization increases insulin exocytosis in pancreatic β cells, through mechanisms which are not clearly understood.^11^

The complex dynamics of the LIMK1-cofilin-actin signaling cascade suggests that pathogenic variants can derail the entire pathway in potentially unpredictable ways. We are the first to report two individuals with heterozygous *LIMK1* missense variants, showing divergent clinical phenotypes, ranging from epileptic encephalopathy to immunodeficiency, and glucose regulation issues. Functional studies in patient-derived cells suggested opposing effects of variants on LIMK1 activity, enabling us to delineate how either decreased or increased LIMK1 activity is linked to aberrant insulin exocytosis, glucose dysregulation, and divergent exocytosis defects.

## Results

### The clinical phenotype of individuals with *LIMK1* variants

In our hospital, we identified two individuals with different *LIMK1 de novo* missense variants, showing divergent clinical phenotypes. Individual 1 is a five-year-old girl who presented with epileptic encephalopathy and developmental delay at the age of eight months. Brain magnetic resonance imaging (MRI) was unremarkable. Despite extensive diagnostic follow-up, no known cause for her symptoms was found. Trio whole exome sequencing (WES) revealed six potential genetic candidates that remained after filtering (Table S1). The *de novo* heterozygous missense variant in *LIMK1* (NM_002314.3: c.1532G>C, p.Gly511Ala) was considered most suspect and followed up in greater detail (Table S1).

**Figure 1:**
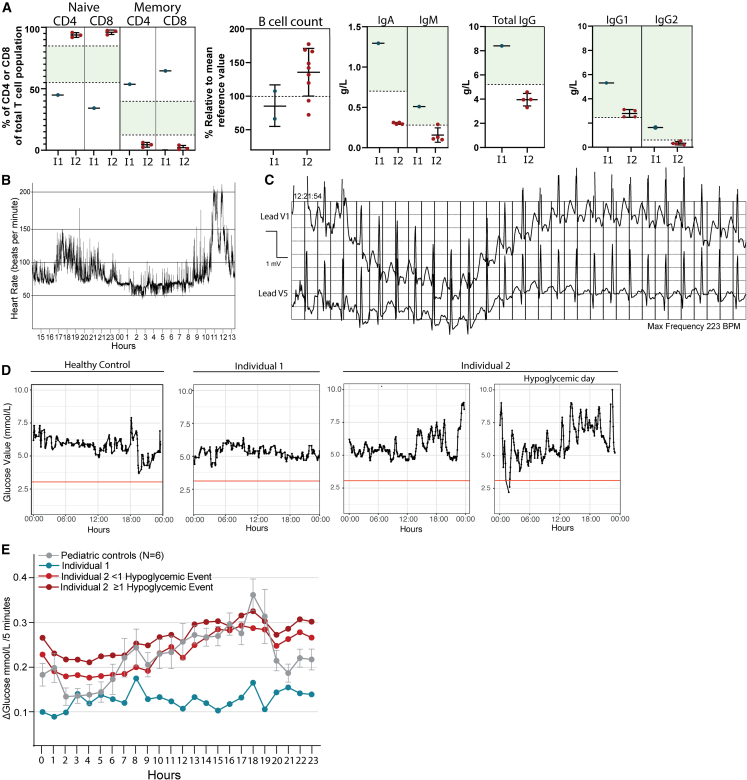
Clinical characteristics of individuals harboring *LIMK1* variants (A) Graphs showing the immunological characteristics of individual 1 and 2. The dots in the graph represent one single observation. Black lines indicate the mean of all observations ± standard deviation (SD). The dotted lines and green planes indicate the reference values in the healthy population. For the percentages of naive and memory cells as part of the total amount of cells, both the lower and upper limit of the reference values are shown. For the immunoglobulins, only the lower limit of the reference value is shown. The B cell counts are shown as a percentage normalized to the mean reference values found in the pediatric population matched on age. (B) Graph showing continuous heart rate measurements of individual 2. On the y axis, beats per minute (BPM) are shown. The graph shows heart rate values on a day with a tachycardic event. The x axis shows the time of day. (C) Holter electrocardiogram (ECG) recording of individual 2 during an episode of inappropriate sinus tachycardia. During the tachycardic event, a maximum heart rate of 223 beats per minute during exercise was observed. (D) Graphs showing glucose values on a representative day (24-h) for one of the pediatric controls, individual 1 and individual 2. For individual 2, two separate graphs are shown. On the left, a normal day is shown, where individual 2 does not experience hypoglycemia. On the right, a “hypoglycemic day” is shown, where individual 2 experiences a nocturnal hypoglycemic event. As can be seen, glucose values remained highly variable throughout the day after a nocturnal hypoglycemic event was recorded. (E) Line graphs showing the average glucose variation throughout the day. For individual 2, two separate lines are shown, one for days where he does not experience hypoglycemic events, and one for days where he experiences one or more hypoglycemic events. Glucose variation was calculated by measuring the absolute difference (delta) between consecutive glucose value measurements (measured every 5 min). Missing values were excluded. The dots indicate the mean glucose variation per hour. The error bars surrounding the gray line indicate the standard deviation for six different pediatric controls. See also Figure S1, Table S2.

Individual 2 presented with a completely different phenotype. During his first year of life, recurrent upper- and lower respiratory infections, diarrhea, and failure to thrive were noted. Decreased levels of immunoglobulins led to the diagnosis of common variable immune deficiency (CVID) (Figure 1A). During childhood, tiredness, exercise intolerance, enuresis nocturna, and hypoglycemic events were noted. The hypoglycemic events were associated with the intake of fast-acting carbohydrates, but were always self-limiting. The hypoglycemia could lead to temporary unconsciousness at times. Additionally, he experienced bouts of sinus tachycardia during exercise or rest that similarly normalized spontaneously (Figures 1B and 1C). Despite extensive diagnostic follow-up, no known cause for his symptoms was found. Trio WES revealed only one potential genetic candidate that remained after filtering, a *de novo* heterozygous missense variant in *LIMK1* (NM_002314.3: c.127G>A; p.Ala43Thr).

Repeated hypoglycemia observed in individual 2 led to continuous glucose measurements (CGM) being performed. In individual 2, hypoglycemic events were frequently recorded: 21% of GCM recorded days (> 900) showed one or more hypoglycemic events, despite receiving hypercaloric intake. Additionally, highly variable glucose values were seen, which were even higher on days where nocturnal hypoglycemic events were recorded (Figures 1D and 1E). In contrast, we found individual 1 showed very little glucose variation compared to pediatric controls (Figures 1D and 1E). However, her ketogenic diet might in part explain this observation (Figure S1).

Immuno-phenotyping revealed decreased percentages of naive T cells and increased levels of memory T cells in individual 1. Intriguingly, individual 2 exhibited exactly opposite values, with high percentages of naive T cells and low numbers of effector memory cells (Figure 1A).

### In-silico predictions

**Figure 2:**
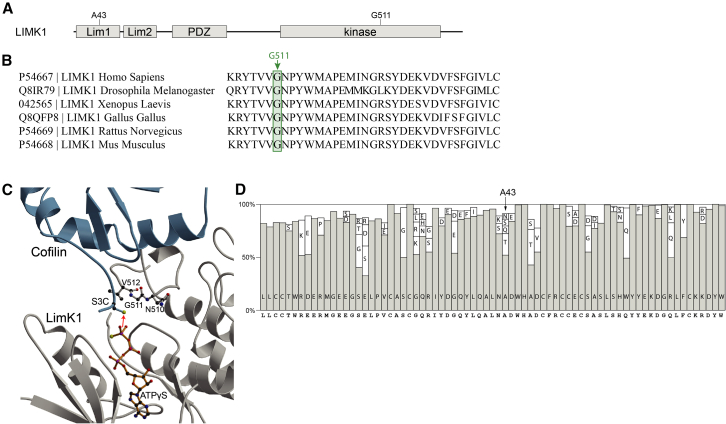
In-silico predictions of the *LIMK1* variants (A) Domain organization of LIMK1. The location of the genetic variants in individual 1 and 2 are indicated. Lim1, Lim domain 1; Lim2, Lim domain 2; PDZ, PDZ domain; kinase, kinase domain. (B) Showing the conservation of the glycine at position 511 among species. The green square and arrow indicate the glycine at position 511. Gly511 and the surrounding residues are highly conserved across species. (C) Ribbon representation of the LIMK1 kinase domain (gray) in complex with cofilin (light blue) and ATPγS, a hydrolysis resistant ATP analogue (orange, ball-and-stick representation). Gly511 and its neighboring residues are shown in ball-and-stick representation. The red arrow indicates the phosphoryl transfer to the substrate. The receiving Ser3 in cofilin was mutated to Cys.^13^^,^^14^^,^^15^ (D) Showing the frequency of different sequence motifs of the LIM1 domain for 370 different species. Amino acid residues with a frequency lower than 4.5% were omitted for clarity of presentation. The most frequent residue is represented as a gray bar. The sequence of human LimK1 is shown underneath.

We predicted the effects of the two different *LIMK1* variants on LIMK1 functionality. While the variant of individual 1 was predicted to result in decreased kinase activity of the protein, the effect of the variant of individual 2 was uncertain, since structural information about the LIM domain is lacking. LIMK1 shows intolerance against loss-of-function, with a probability of loss of function intolerance (pLI) score of 1.0 and o/e ratio of 0.09.^12^ The *LIMK1* variant of individual 1 is located at a highly conserved residue in the kinase domain: c.(1532G>C); p.(Gly511Ala) (Figures 2A and 2B). It resides closely to the site of phosphorylation for LIMK1 activation (Thr-508).^6^ The variant has not been reported in GnomAD. PolyPhen-2^13^ predicts this variant to be probably damaging (0.995), with a combined annotation dependent depletion (CADD) score of 26.9.^16^ Gly511 resides within the catalytic domain and is part of the activation segment, where the transfer of the phosphate from ATP to the substrate takes place.^17^ The backbone conformation of Gly511 adopts ϕ- and Ψ-angles that are impossible for other amino acids to adopt (Figure 2C). The second individual harbored a variant in the first LIM domain: c.(127G>A); p.(Ala43Thr) (Figure 2A). The Ala43Thr variant has been reported with an allele frequency of 0.000009. PolyPhen-2 predicts p.Ala43Thr to be benign, and the variant has a CADD score of 13.4. Ala43 is substituted for threonine in many species (Figure 2D). Interestingly, a shorter LIMK1 isoform exists, that lacks major parts of the first Lim domain including Ala43. It is not clear to what extent this isoform is biologically relevant. While structural information about the LIM domains is lacking, the LIM domains are known to act as an auto-inhibitor of the protein.^7^ Potentially, a destabilizing effect of the Ala43Thr on the Lim domain variant could result in increased activity of LIMK1.

### Functional assay results

We set out to study the functional effects of *LIMK1* variants in patient-derived fibroblasts. We studied LIMK1 availability, cofilin phosphorylation and actin polymerization, and found divergent effects of the different variants on these assays. We included a patient with heterozygous *LIMK1* deletion for comparison. The patient harbored a large deletion encompassing the entire *LIMK1* gene and the last exon of elastin gene (*ELN)*. The patient showed verbal delay and learning disability, together with ELN-related phenotypic features, such as supravalvular pulmonary- and aortic-stenosis.

**Figure 3:**
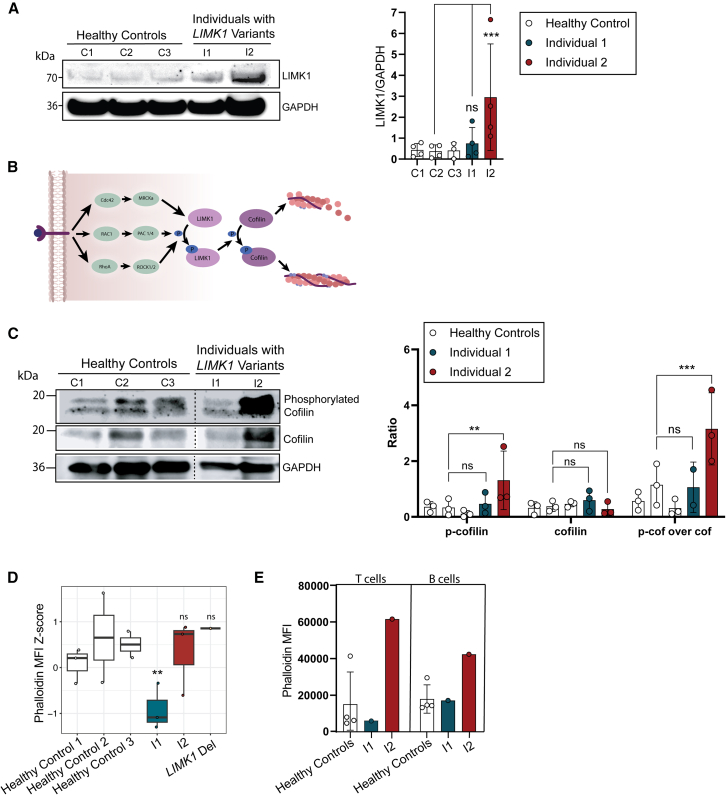
Functional assessment of the *LIMK1* variants in fibroblasts and lymphocytes (A) Representative western blot showing LIMK1 protein levels in fibroblasts derived from three healthy controls, individual 1 (I1) and individual 2 (I2). Glyceraldehyde-3-fosfaat dehydrogenase (GAPDH) was used as housekeeper. The bar graphs show the ratio of LIMK1 band intensity normalized to GAPDH. For the bar graph, three independent western blot experiments were used (Figure S2). Each bar represents the mean of one patient or healthy control, each dot represents one technical replicate. The bar graphs show the mean values ± SD. Statistics were calculated using linear mixed models. *p* values ∗*p* < 0.05; ∗∗*p* < 0.01, ∗∗∗*p* < 0.001, ∗∗∗∗*p* < 0.0001 were considered significant. (B) Graphical representation of LIMK1 and its downstream functions. On the far right, polymerized actin strands are visualized in red. (C) Representative western blot showing cofilin and phosphorylated cofilin (Ser3) protein levels in fibroblasts derived from three healthy controls and individual 1 and 2. GAPDH was used as housekeeper. The bar graph shows the ratio of cofilin or phosphorylated cofilin over GAPDH. For the bar graph, three independent western blot experiments were used (Figure S2). Each bar represents one biological replicate, each dot represents one technical replicate. The bar graphs show the mean values ± SD. Statistics were calculated using linear mixed models. *p* values ∗*p* < 0.05; ∗∗*p* < 0.01, ∗∗∗*p* < 0.001, ∗∗∗∗*p* < 0.0001 were considered significant. (D) Bar graphs showing the mean fluorescence intensity (MFI) ±SD from multiple flow cytometry experiments in fibroblasts. The gating strategy can be found in Figure S4. Statistics were calculated using linear mixed models. *p* values ∗*p* < 0.05; ∗∗*p* < 0.01, ∗∗∗*p* < 0.001, ∗∗∗∗*p* < 0.0001 were considered significant. See also Figure S6. (E) Bar graphs showing the mean fluorescence intensity (MFI) ±SD for four different healthy controls (*N* = 4) and for individual 1 and 2 (*N* = 1 each). The histograms used to calculate the median fluorescence intensity can be found in Figure S5. The gating strategy can be found in Figure S3.

Overall, LIMK1 expression varied between donors, but average LIMK1 protein levels in fibroblasts of individual 1 and the patient with *LIMK1* hemideletion were similar compared to healthy control fibroblasts (Figures 3A and S2). In contrast, fibroblasts of individual 2 showed significantly increased LIMK1 protein levels (Figures 3A and S2). To study LIMK1 functionally, we assessed phosphorylated cofilin protein levels in fibroblasts (Figures 3B and S2). We found individual 1 showed normal phosphorylated cofilin over cofilin ratios compared to healthy controls (Figure 3C). In support of overactive LIMK1, we found increased levels of phosphorylated cofilin and phosphorylated cofilin (*p*-cofilin) over cofilin ratio’s in fibroblasts of individual 2 (Figure 3C). In the individual with heterozygous *LIMK1* deletion, we found lower cofilin protein levels leading to a higher *p*-cofilin over cofilin ratio as well (Figure S2). The increased levels of LIMK1 protein and LIMK1 activity could indicate that the Ala43Thr variant leads to increased half-life and stability, potentially due to increased phosphorylation by LIMK1 itself or upstream kinases.^9^

Next, we measured levels of polymerized actin in fibroblasts and human peripheral blood mononuclear cells (PBMCs) using flow cytometry. Here, we found differences between fibroblasts of individual 1 and the patient with *LIMK1* hemideletion, as individual 1 showed decreased actin polymerization while the patient with *LIMK1* hemideletion did not (Figures 3D and S4). In contrast, we found increased levels of actin polymerization in fibroblasts of individual 2, albeit not significant (Figure 3D). Subsequent assessment of phalloidin distribution using microscopy showed bright phalloidin spots that could reflect local actin aggregates (Figure S6). In T and B cells, we found no significant difference in actin polymerization with flow cytometry in individual 1, however, the distribution seemed different between cells compared to healthy controls (Figures 3E and S3). In individual 2, we observed a trend toward increased levels of actin polymerization in T and B cells (Figures 3E and S3).

Together, these results suggest divergent effects of *LIMK1* variants. While absolute cofilin phosphorylation levels were not altered in fibroblasts of individual 1, cofilin phosphorylation dynamics could have been affected, illustrated by decreased actin polymerization. Conversely, in individual 2, the increased cofilin phosphorylation and actin polymerization suggest there is increased LIMK1 activity in this individual.

### Translation of cellular defects to the clinical phenotype

Given the aberrant glucose regulation observed in individual 2, and the role of LIMK1 in insulin exocytosis,^18^ we studied INS-1 cells, that release insulin upon stimulation with glucose, overexpressing either wild type (WT)-LIMK1 or LIMK1 harboring Gly511Ala (individual 1) or Ala43Thr (individual 2) variants. Again, we found divergent effects of the *LIMK1* variants on exocytosis dynamics, although insulin secretion was increased for both variants.

**Figure 4:**
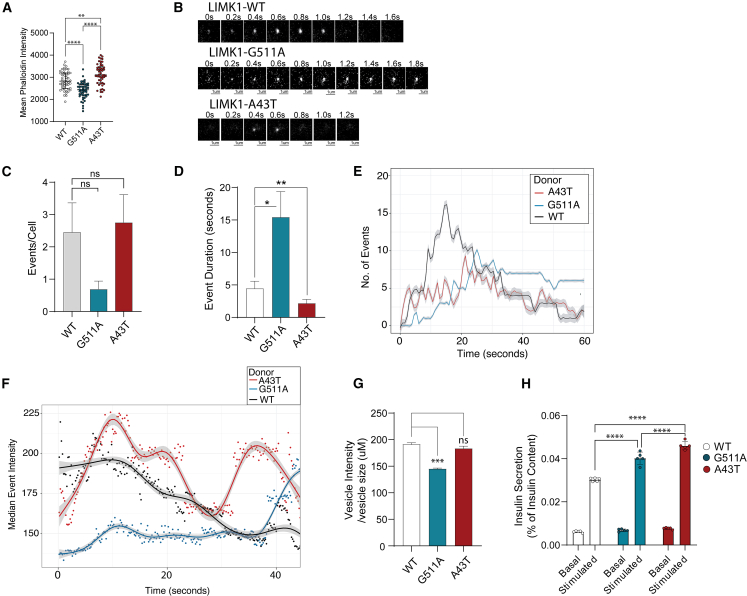
Insulin exocytosis measurements in INS-1 cells after the introduction of different *LIMK1* constructs Exocytotic events were visualized by introducing neuropeptide Y (NPY)-pHluorin (a pH-sensitive GFP that fluoresces under higher pH) in cell lines. After stimulation of INS-1 cells with medium containing high levels of glucose/potassium, vesicle exocytosis was measured for 60 s. Images were taken every 0.2 s. The experiments were performed in triplicate. The results of one representative experiment are shown. (A) Phalloidin staining intensity in INS-1 cells overexpressing human influenza hemagglutinin (HA)-WT-LIMK1 or mutant LIMK1. Each dot reflects the value of one cell, the lines reflect the median with interquartile range (IQR) 25–75. Statistics were calculated with one-way analysis of variance (ANOVA) followed by Turkey’s multiple comparison test. *p* values ∗*p* < 0.05; ∗∗*p* < 0.01, ∗∗∗*p* < 0.001, ∗∗∗∗*p* < 0.0001 were considered significant. See also Figure S6. (B) Showing representative images of exocytotic events in INS-1 cells expressing either the LIMK1-WT, LIMK1-Gly511Ala or LIMK1-Ala43Thr construct. (C) Bar graph showing the mean number of exocytosis events for the three INS-1 cell lines normalized to the number of cells ±SD. For each cell line, approximately 30 cells were analyzed (WT = 31, Ala43Thr = 36, Gly511Ala = 29). Statistics were calculated with one-way ANOVA followed by Turkey’s multiple comparison test. *p* values ∗*p* < 0.05; ∗∗*p* < 0.01, ∗∗∗*p* < 0.001, ∗∗∗∗*p* < 0.0001 were considered significant. (D) Bar chart showing the mean duration of complete vesicle secretion for each exocytosis event. The bar reflects the mean duration for all events per donor ±SD. 2% of outliers were removed using the robust regression and outlier removal (ROUT) method.^19^ Statistics were calculated using one-way ANOVA followed by Dunn’s test. *p* values were adjusted for multiple comparisons. *p* values ∗*p* < 0.05; ∗∗*p* < 0.01, ∗∗∗*p* < 0.001, ∗∗∗∗*p* < 0.0001 were considered significant. (E) Line graph showing the number of exocytosis events observed at each timepoint (Event rate/second). INS-1 cells expressing the LIMK1-Ala43Thr construct showed faster vesicle secretion, therefore less exocytosis events were observed at each timepoint, but the number of events fluctuated heavily over time. INS-1 cells expressing the Gly511Ala variant showed slower vesicle secretion, and events started significantly later after potassium/glucose stimulation. (F) Line graph showing the median intensity of vesicles over time. Intensity values were normalized for vesicle size (total area). Most events lasted between 1 and 40 s, and therefore the x axis was adjusted accordingly. Every 0.2 s, the median intensity of the events present at that timepoint was calculated. The values were plotted using a generalized additive model (GAM) with a cubic spline basis (bs = “cs”). The gray shades surrounding the lines represent the 95% confidence intervals of the model. The dots represent raw data points. (G) Bar chart showing the median intensity values normalized for vesicle size (total area) per vesicle. The bar shows the mean value per donor ±SD. Statistics were calculated using one-way ANOVA followed by Turkey’s multiple comparison test. *p* values ∗*p* < 0.05; ∗∗*p* < 0.01, ∗∗∗*p* < 0.001, ∗∗∗∗*p* < 0.0001 were considered significant. (H) Bar graph showing the basal and stimulated insulin secretion for INS-1 cells overexpressing the different *LIMK1* variants. Insulin was measured after 2 h in low glucose/potassium or high glucose/potassium buffer. The insulin values measured are normalized to the intracellular insulin content. Bars reflect the mean value for five biological replicates ±SD. Statistics were calculated using one-way ANOVA followed by Turkey’s multiple comparison test. *p* values ∗*p* < 0.05; ∗∗*p* < 0.01, ∗∗∗*p* < 0.001, ∗∗∗∗*p* < 0.0001 were considered significant.

We found different LIMK1 protein levels were reached after overexpression of the WT-LIMK1, LIMK1-Gly511Ala, and LIMK1-Ala43Thr variants. (Figure S7). In concurrence, we found decreased phalloidin intensity in rat insulinoma (INS-1) cells overexpressing the Gly511Ala variant, and increased phalloidin intensity in INS-1 cells overexpressing the Ala43Thr variant (Figure 4A) compared to INS-1 cells ectopically expressing WT-LIMK1. Visual inspection of the LIMK1-Gly511Ala expressing cell line revealed slow and dim exocytosis events, while the LIMK1-Ala43Thr expressing INS-1 cell line seemed to have a flashy appearance (Figure 4B, Videos S1, S2, S3, S4, S5, and S6). Upon quantification, we found LIMK1-Gly511Ala overexpression showed a trend toward decreased exocytotic events (Figure 4C). In contrast, INS-1 cells expressing the Ala43Thr variant showed a trend toward increased exocytotic events after glucose stimulation compared to INS-1 cells ectopically expressing WT-LIMK1 (Figure 4C). The entire process of vesicle secretion in INS-1 cells expressing LIMK1-Gly511Ala lasted significantly longer (Figure 4D). Additionally, exocytosis commenced significantly later after glucose stimulation, and new events continued to emerge throughout the measurement period, while in the cells expressing WT-LIMK1, new events emerged primarily in the first 20 s (Figure 4E). In contrast, exocytotic events in INS-1 cells expressing Ala43Thr were significantly shorter, started more rapidly after glucose stimulation and fluctuated heavily over time (Figures 4D–4F). For INS-1 cells expressing the Gly511Ala-LIMK1 construct, we found that the visually dim appearance of the vesicles secreted correlated with significantly decreased total intensity values over time (Figure 4F) and overall intensity per cell (Figure 4G). INS-1 cells expressing the LIMK1-Ala43Thr variant showed a high intensity peak which fluctuated throughout the measurement period, in line with its visually flashy appearance (Figure 4F). However, total intensity per cell compared to WT-LIMK1 was not significantly altered (Figure 4G). These differences in intensity could be due to either lower pH of the vesicles or decreased amount of cargo (insulin). To address this, we measured the amount of insulin secreted by INS-1 cells with an enzyme-linked immunosorbent assay (ELISA) after 2 h of glucose/potassium stimulation. Surprisingly, we found that both variants (Gly511Ala and Ala43Thr) lead to increased insulin secretion compared to WT-LIMK1 (Figure 4H). While this seems controversial for the Gly511Ala-LIMK1 expressing cell line, it might correlate with a slower onset but prolonged continuation of exocytosis after glucose stimulation, leading to an overall increase in insulin secretion over time.

Video S1. Showing representative exocytotic events in INS-1 cells stably expressing WT-LIMK1, related to Figure 4

Video S2. Showing representative exocytotic events in INS-1 cells stably expressing G511A-LIMK1, related to Figure 4

Video S3. Showing representative exocytotic events in INS-1 cells stably expressing A43T-LIMK1, related to Figure 4

Video S4. Unprocessed video showing the entire cell INS-1 cells stably expressing WT-LIMK1 (Video S1), Related to Figure 4

Video S5. Unprocessed video showing the entire cell INS-1 cells stably expressing G511A-LIMK1 (Video S2), related to Figure 4

Video S6. Unprocessed video showing the entire cell INS-1 cells stably expressing A43T-LIMK1 (Video S3), related to Figure 4

In conclusion, we found that overexpression of the Gly511Ala variant (individual 1) in INS-1 cells led to decreased exocytosis, prolonged event duration, and higher overall insulin secretion. In contrast, the Ala43Thr variant demonstrated faster event duration with higher peak intensity, yet similarly resulting in increased insulin secretion.

## Discussion

We present two individuals harboring *LIMK1* variants with divergent clinical phenotypes and propose that these differences align with divergent LIMK1 activity. Individual 1 showed developmental delay and epilepsy, and decreased actin polymerization in patient-derived fibroblasts suggested decreased LIMK1 kinase activity. Upon overexpression of this variant, significantly slower exocytosis was found in insulin secreting (INS-1) cell lines. Conversely, individual 2 showed CVID, disordered glucose regulation, and sinus tachycardia. In fibroblasts, increased LIMK1 availability and cofilin phosphorylation were seen. Upon overexpression of the Ala43Thr variant in INS-1 cells, rapid, and flashy exocytosis events were observed, in contrast to those seen with WT-LIMK1. Intriguingly, both variants led to increased overall insulin secretion. Together, these results indicate that heterozygous variants in *LIMK1* may give rise to neurological and hormonal disturbances, potentially through altered exocytosis dynamics.

The first aim of this study was to establish whether *LIMK1* variants were pathogenic and give rise to a novel syndrome. The variant of individual 1 was predicted to disrupt the local fold and the highly conserved catalytic site of LIMK. While normal levels of phosphorylated cofilin might argue against pathogenicity, the significantly decreased levels of polymerized actin—absent in heterozygous loss of LIMK1—suggest that cofilin phosphorylation dynamics could have been affected. The severity of the clinical phenotype found in individual 1 suggests the variant exerts effects beyond the mere loss of one active *LIMK1* copy alone, although this remains to be verified. Putatively, the mutant *LIMK1* binds the unaffected *LIMK1* copy through homo-dimerization.^9^ Alternatively, LIMK1-Gly511Ala might mimic the expression pattern of a specific LIMK1 isoform (ENST00000435201.1) lacking the catalytically active site. For LIMK2, a similar isoform (LIMK2d) has been identified, and was found to negatively affect neurite outgrowth in rats.^20^ This catalytically dead isoform might exert specific functions in the regulation of the entire LIMK1 pool. The effect of a catalytically dead LIMK1 isoform might be exaggerated locally, when LIMK1 works close to saturation to allow for rapid actin depolymerization, which is facilitated through the many feedback loops within the LIMK1-cofilin-actin pathway.^5^

The variant of individual 2 was associated with significantly increased LIMK1 and phosphorylated cofilin levels, suggesting that the Ala43Thr variant could affect the auto-inhibitory function of the first LIM domain. Interestingly, a shorter LIMK1 isoform (ENST00000538333.3) exists, lacking part of the first LIM domain, including the Ala43Thr variant. While detailed information about this shorter isoform is lacking, it could be used to regulate overall LIMK1 expression by converging LIMK1 to a more open confirmation. Exploring the clinical relevance of these isoforms across different tissues and their impact on LIMK1 activity could provide an exciting avenue for further research.

LIMK1 and cofilin are both activated upon glucose stimulation in pancreatic β cells (MIN6-K8).^18^ The second phase of insulin secretion is characterized by a continuously high state of LIMK1- and cofilin phosphorylation, that favors the presence of F-actin, allowing insulin vesicles further away from the plasma membrane to be recruited.^18^ Reduced expression of cofilin leads to a more rapid induction of insulin secretion, which concurs with the rapid burst of exocytosis observed with LIMK1-Ala43Thr overexpression (the variant of individual 2).^18^ In contrast, expression of a constitutively active form of cofilin, leading to a depletion of the G-actin pool preventing actin polymerization, slows down the kinetics of insulin secretion,^18^ potentially through a mechanism where distant vesicles cannot be recruited. Similarly, the LIMK1-Gly511Ala variant could have limited the recruitment of distant vesicles, leading to the decreased number of exocytotic events observed. However, the various roles of actin in several exocytotic processes (endocytosis, vesicle recruitment, docking, etc.) make it challenging to pinpoint specifically which steps would be most affected.

Intriguingly, expression of both the Gly511Ala and the Ala43Thr variant were associated with increased insulin secretion after 2 h. It is remarkable how these opposing effects can lead to a net increased insulin exocytosis in INS-1 cells, which may reflect the contradictory findings on the role of actin polymerization in insulin exocytosis dynamics reported in recent years. Additionally, a recent study on the same two individuals with *LIMK1* variants found that both variants disrupted cell cycle progression in a similar way, resulting in an increased proportion of cells in the S/M phase.^21^ Nonetheless, while the increased insulin exocytosis in INS-1 cells does not necessarily concur with total insulin secretion in the human body, it is striking that the observed exocytosis defects and increased insulin secretion are not associated with glucose regulation issues in individual 1. One possible explanation is that glucose regulation problems are more likely to occur when insulin secretion is rapid and erratic, rather than when it is slow. Slower insulin secretion might be offset by slower glucagon secretion, resulting in minimal impact on glucose regulation overall. However, the slower exocytosis might be far more destructive to organs that require rapid kinetics, such as neurons, which could concur with the predominantly neurological phenotype of individual 1. Regarding individual 2, the hypoglycemic events observed shortly after ingestion of fast-acting carbohydrates resemble the rapid and uncontrolled insulin exocytosis observed in INS-1 cells after glucose stimulation, suggesting a functional link. Additionally, other characteristics of his clinical phenotype, such as enuresis nocturna and episodic tachycardia, make it tempting to speculate that uncontrolled exocytosis of vasopressin or catecholamines could similarly play a role in underlying pathophysiology.

The epileptic encephalopathy in individual 1 prompts consideration of aberrant neurotransmitter exocytosis as a potential contributor, given the established link between epilepsy and aberrant neurotransmitter exocytosis.^22^ Drawing similarities from our exocytosis, one would expect that the Gly511Ala variant would lead to slower exocytosis dynamics while the Ala43Thr variant leads to more rapid neurotransmitter release. However, in line with our insulin measurements showing increased insulin secretion for both variants, predicting whether overall neurotransmitter secretion is increased or decreased in neurons remains challenging. While *LIMK1* knockout mice show disrupted neurotransmitter exocytosis, they do not present with epilepsy.^23^ Cofilin knockout mice do show epilepsy,^24^ and actin depolymerizing factor (ADF)/N-cofilin (CFL1) double knockout mice have impaired neuronal vesicle recruitment and exocytosis, resulting in decreased glutamate secretion in neuronal synapses.^25^ The inconsistencies in the relationship between exocytosis and epilepsy might be due to the complex and dynamic nature of vesicle endocytosis, exocytosis, and feedback mechanisms required by neurons that are not optimally reflected in *in vitro* experimental setups. Alternatively, the pathophysiology of seizures could be related to neuronal migration deficits or altered synapse formation rather than exocytosis,^26^ since *LIMK1* knockout mice show clearly altered dendritic spine morphology.^23^ In support of this, patients with pathogenic variants in cytoplasmic actin genes *ACTB* and *ACTG1* similarly show seizures, which are thought to originate through neuronal migration deficits.^27^ Studying the pathophysiology of seizures and its relation to the many functions of actin could provide exciting avenues for further research.

In this manuscript, we focused primarily on the effect of LIMK1 dysfunction through its interaction with cofilin and actin. However, several other proteins are suggested to bind to LIMK1,^28^^,^^29^^,^^30^^,^^31^^,^^32^ although the evidence for their interaction is not as well-established. Since we did not assess cofilin phosphorylation in the INS-1 cells after expressing the different *LIMK1* variants, we cannot exclude the possibility that some of the phenotypes observed in individuals with *LIMK1* variants are mediated through altered binding of other proteins than cofilin. LIMK1 has been shown to interact with fascin-1/actin and paxillin-mediated Rho activation.^33^^,^^34^ Since both of these proteins mediate actin polymerization, altered binding of LIMK1 with fascin-1 or paxillin could have similarly led to the observed differences in actin polymerization. Moreover, recent work has shown that both LIM domains of LIMK1 are required for LIMK1 to bind to keratin.^35^ In addition, LIMK1 is thought to stimulate cAMP response element-binding protein (CREB) activity, and reduced CREB activity sensitizes the heart to stress-induced ventricular arrhythmias by altering ion channel regulation and action potential duration.^36^^,^^37^^,^^38^ Thus, altered activity of CREB might be able to explain the episodic sinus tachycardia observed in individual 2.

The phenotypic features in individuals with *LIMK1* variants align with those seen in other actinopathies,^3^ where immunodeficiency is a common manifestation. Similarly, developmental delay and seizures have been observed in actinopathies, although to a lesser extent.^39^^,^^40^^,^^41^^,^^42^^,^^43^ While many actin-modifying proteins have been linked to insulin exocytosis at the cellular level,^44^^,^^45^^,^^46^^,^^47^ clinical glucose dysregulation has been reported only marginally.^14^^,^^15^ Potentially, this is due to the fact that actin-mediated glucose dysregulation only manifests itself when studied with continuous glucose monitoring, or alternatively, that LIMK1 dysfunction exerts more pronounced effects on glucose homeostasis compared to other actin-modifying genes. The unique effect of altered LIMK1 expression could be due to specific interactions of LIMK1 with other proteins, for example through altered expression of the insulin receptor that is dependent on LIMK1 expression.^48^ However, this initial report of an actinopathy with a distinct endocrinological phenotype, combined with differing exocytosis dynamics in INS-1 cells, points to the crucial role of LIMK1-mediated actin remodeling in exocytosis. The individuals described in this study suggest that tight regulation of LIMK1 activity is essential to warrant appropriate actin tuning.

### Limitations of the study

The findings of this study, based on evidence from single patients, call for cautious interpretation of the proposed effects on LIMK1 functionality. Validating these results in additional patients would aid in determining whether other (genetic) factors contributed and would provide a clearer assessment of the correlation between LIMK1 variation and clinical- and cellular phenotypes. Since LIMK1 expression differs between individuals due to LIMK1 promotor region variants, the differences in LIMK1 expression observed could be exaggerated or caused by this kind of genetic variation.^49^ In addition, performing additional experiments, comparing the LIMK1-Ala43Thr and Gly511Ala variants to LIMK1-WT and an empty vector, could help verify whether these variants indeed exert dominant negative effects. Finally, studying LIMK1 mRNA levels and protein degradation can help determine whether the LIMK1-Gly511Ala variant indeed results in decreased stability of the LIMK1 protein pool through decreased *trans*- or autophosphorylation.

## Resource availability

### Lead contact

Further information and requests for resources and reagents should be directed to and will be fulfilled by the lead contact, Peter M. van Hasselt.

### Materials availability

This study did not generate new unique reagents.

### Data and code availability

• *LIMK1* genetic variants data have been deposited at Clinvar and are publicly available at the date of publication. The accession numbers are: ClinVar: SCV004190115 and ClinVar: SCV005068253
• This paper does not report original code.
• Any additional information required to reanalyze the data reported in this paper is available from the lead contact upon request

## Acknowledgments

We thank the Dr. Arvan lab at University of Michigan for gifting the INS-1 cells used in this study. We also thank the Dr. Edwards lab at UCSF for gifting us the psPAX, pVSVS, NPY-pHluorin, and NPY-sfCherry vectors.

## Author contributions

Conceptualization: I.J.J.M., C.S.A., and P.M.v.H.; methodology: I.J.J.M., C.S.A., and P.M.v.H.; investigation: I.J.J.M., T.C., H.R., S.J.V., A.A.V.S., A.C.B., A.G., B.v.d.Z., I.M.D.L., J.C.G., K.L.I.v.G., K.K., C.S.A., and P.M.v.H.; visualization: I.J.J.M., T.C., C.S.A., and P.M.v.H.; supervision: C.S.A. and P.M.v.H.; writing – original draft: I.J.J.M. and P.M.v.H.; writing – review and editing: I.J.J.M., H.R., S.J.V., A.A.V.S., A.C.B., B.v.d.Z., K.L.I.v.G., K.K., C.S.A., and P.M.v.H.

## Declaration of interests

The authors declare that no conflict of interest exists.

## STAR★Methods

### Key resources table

**Table:** 

REAGENT or RESOURCE	SOURCE	IDENTIFIER
**Antibodies**		

Phosphorylated-Cofilin	Cell Signaling	Cat#: CST 3313T
Cofilin	Cell Signaling	Cat#: CST 5175S
LIMK1	Enzo life sciences	Cat#: ALX-803-343-C100
GAPDH	Novus Biologicals	Cat#: NB300-221, RRID: AB_1007762
Mouse Anti-a-Tubulin	Millipore	Cat#: T5168, RRID: AB_477579
Live/dead fixable viability dye	Thermofisher	Cat#: 65-0866-18
CD3	Biolegend	Cat#: 100216, RRID: AB_493697
CD19	Sony Biotechnology	Cat#: 2111140
DRAQ5	Biolegend	Cat#: 424101
Phalloidin	Sigma Aldrich	Cat#: P1951, RRID: AB_2315148
Goat anti rabbit	Dako	Cat#: P044801-2
Rabbit anti mouse	Dako	Cat#: P0260, RRID: AB_2636929
Rat anti-HA	Roche	Cat#: 11867423001, RRID: AB_390918
Phalloidin-iFluor 488 Reagent	Abcam	Cat#: ab176753

**Biological samples**		

Patient derived fibroblasts (individual 1 and 2) and healthy control fibroblasts	Wilhelmina Children’s Hospital	N/A
Patient derived PBMCs and healthy donor PBMCs	Wilhelmina Children’s Hospital	N/A
Patient with LIMK1/ELN hemideletion	Center Hospitalier Universitaire (CHU) Dijon	N/A

**Chemicals, peptides, and recombinant proteins**		

FBS	Sigma	Cat #F7524
DAPI Fluoromount-G	Southern Biotech	Cat#: 0100-20
Radioimmunoprecipitation assay (RIPA) buffer	Sigma Aldrich	Cat#: R0278-50mL
Polyvinylidene fluoride (PVDF) membranes	Millipore Sigma	Cat#: IPFL00010
DMEM/F-12	Thermofisher	Cat#:11765054
HAM’s F-12 Nutrient Mix	Thermofisher	Cat#: 11765054
RPMI	GenClone	Cat#: 25-506
Sodium Pyruvate	HyClone	Cat#: SH30239.01
BD Cytofix/Cytoperm Fixation/Permeabilization Kit	BD Biosciences	Cat#: 554714
StemPro Accutase Cell Dissociation Reagent	Thermofisher	Cat#: A1110501
HEPES	GoldBio	Cat#: H-400-1
FBS (for INS-1 cells)	GenClone	Cat#: 52-525
Penicillin-Streptomycin	Thermofisher	Cat#: 15140122
Trypsin	Thermofisher	Cat#: 15400054
TrypLE	Thermofisher	Cat#: 12604021
Phosphatase inhibitor cocktail	Merck	Cat#: P5726-1ML
Ficoll Paque	Sigma	Cat#: GE17-1440-02
Protease inhibitor cocktail	Fisher Scientific	Cat#: 10516495
Puromycin	GoldBio	Cat#: P-600-100
PEI	Sigma	Cat#: 03880
Gibson Assembly Master Mix	New England Biolabs	Cat#: E2611L
Lipofectamine 2000	ThermoFisher	Cat#: 11668027
Glass Coverslips	Warner Instruments	Cat#: 64-0735

**Critical commercial assays**		

BCA assay kit	Thermofisher	Cat#: 23225
Cytofix/Cytoperm Fixation Permeabilization kit	BD Biosciences	Cat#: 554714
E.Z.N.A. Plasmid DNA Mini Kit I	Omega Bio-Tek	Cat#: D6922-04
Rat Insulin ELISA Kit	ThermoFisher	Cat#: ERINS

**Deposited data**		

LIMK1 genetic variants	ClinVar	ClinVar: SCV004190115 and ClinVar: SCV005068253

**Experimental models: Cell lines**		

INS-1 cells	Gifted by Dr. Arvan lab at U of Michigan	N/A

**Recombinant DNA**		

pLenti-CMV-puro plasmid (pLenti-CMV-MCS-GFP-SV-puro)	Addgene	Plasmid # 73582; http://n2t.net/addgene:73582; RRID:Addgene_73582
psPAX	Gifted by Dr. Edwards lab at UCSF	N/A
pVSVS	Gifted by Dr. Edwards lab at UCSF	N/A
NPY-pHluorin	Dr. Edwards lab at UCSF	N/A
NPY-sfCherry3	Cloned from backbone pCAGGS gifted by Dr. Edwards lab at UCS. Insert is based on the sequence of Feng et al.,^50^	N/A

**Software and algorithms**		

R 4.4.1	N/A	https://www.rstudio.com
RStudio 2024-04-2	N/A	https://www.rstudio.com
GraphPad Prism 10	N/A	https://www.graphpad.com/
ImageJ 2.14.0	N/A	https://imagej.net/ij/
FlowJo	N/A	https://www.bdbiosciences.com/
FACS DIVA software	N/A	https://www.bdbiosciences.com/
Adobe Illustrator	N/A	www.adobe.com
Image Lab software 5.1	N/A	https://www.bio-rad.com/
MolScript	N/A	https://pekrau.github.io/MolScript/
Raster3D	N/A	http://skuld.bmsc.washington.edu/raster3d/
FiloQuant	N/A	https://doi.org/10.1083/jcb.201704045
EVOS XL Core Imaging System	ThermoFisher	N/A
Confocal SP8	Leica	N/A
LSRFortessa High-Parameter Flow Cytometer	BD Biosciences	N/A

**Other**		

4-20% MP TGX Stain-Free Gel 10W 50ul ×10	BioRad	4568094

### Experimental model and study participant details

#### Human participants

Individual 1 is a five-year old girl, while individual 2 is a 16-year old boy. The patient with *LIMK1* hemideletion is a 9-year old boy. All individuals with *LIMK1* variants have high socioeconomic status. All patients are Caucasian and of Northern European descent. The healthy fibroblast lines used in this study are 1 years old (*N* = 2), 3 years old (*N* = 3), and 23 years old, and consist of four females and two males. Written informed consent was obtained from participants (>16 years of age), from participants and their legal guardians together (12–16 years) or from the legal guardians (<12 years). Fibroblast cells and Peripheral Blood Mononuclear Cells were collected for diagnostic purposes, and written informed consent of parents and/or patients (P1 and P2) were obtained to store and use residual material of these biopsies in the Wilhelmina Children’s Hospital Metabolic biobank (TCBio 19–489/B, MET22-484). Healthy adult donor Peripheral Blood Mononuclear Cells (PBMCs) (*N* = 4) were obtained through the Minidonor Services, an ethics review board-approved blood donation facility at the UMC Utrecht (TCBio 18–774) Healthy fibroblast lines were recruited through the Wilhelmina Children’s Hospital metabolic biobank. Informed consent and fibroblast lines from CHU Dijon were included under NCT03287193. Rat insulinoma cells (INS-1) were used to assess exocytosis and insulin secretion. The LIMK1 variants were submitted to the ClinVar repository under accession numbers ClinVar: SCV004190115 and ClinVar: SCV005068253. All procedures performed in studies involving human participants were in accordance with the Helsinki Declaration (as revised in 2013) and the ethical standards of the Medisch Ethische Toetsingscomissie (METC) in The Netherlands: TCBio 19–489/B, MET22-484, TCBio 18–774, and the ethical standards of the ANSM (Agence Nationale de Sécurité du Médicament et des Produits de Santé): 2016-A01347-44 and the The Ethics committee East 1 (Comité de Protection des Personnes Est1), study registered under the file N° 2016/38.

#### *In-vitro* cell culture

PBMCs were derived by blood withdrawals in Ethylenediaminetetraacetic acid (EDTA) tubes of individual 1 and 2 and healthy control donors (*N* = 4, ages: 27, 32, 56, 58, all females). PBMCs were isolated using Ficoll and immediately frozen after harvesting. On the day of the assay, PBMCs were thawed and stained immediately for flow cytometry. For fibroblast cultures, forearm punch biopsies from individual 1, individual 2, the patient with hemizygous LIMK1 deletion and healthy control subjects were cut into small pieces, and the epidermal layer was removed. The small dermal biopsy parts were incubated in fibroblast culture medium HAM’s F-12 Nutrient Mix supplemented with 20% fetal bovine serum (FBS), penicillin [100 UI/mL] and streptomycin [100 μg/mL]) in a humidified incubator at 37°C and 5% CO2. As soon as fibroblasts were growing from the biopsies, medium was changed to HAM’s F-12 nutrient mix with 10% FBS and fibroblasts were passaged every 3–4 days using trypsin-EDTA. INS-1 cells were kindly provided by the Dr. Arvan lab at U of Michigan.

### Method details

#### Exome sequencing

The variants of the two individuals were identified through trio WES. Exomes were enriched using Agilent SureSelect XT Human All Exon kit V5 and sequenced on a HiSeq sequencing system (Illumina). Reads were aligned to hg19 using Burrows–Wheeler Aligner. Variants were called using Genome Analysis Toolkit Variant Caller and annotated, filtered, and prioritized using the Bench Next Generation Sequencing Lab platform (Agilent-Cartagenia, Leuven, Belgium) and/or an in-house designed “variant interface” and manual curation. The minimal coverage of the full target was >15 × 99%. All common polymorphisms with a minor allele frequency (MAF) higher than 0.5% were filtered out using several public databases.^51^^,^^52^^,^^53^^,^^54^^,^^55^ Detailed information about the filtering steps and population databasescan be found in recent work of Snoek et al., 2022.^56^ Alamut (Version 2.4), that integrates multiple splice site prediction algorithms, was used to predict the effect of variants on pre-mRNA splicing. For both individuals, all variants that remained after filtering steps are show in Table S1. The variants were sorted based on their likelihood to cause the observed patient phenotype, by employing in-silico prediction models that consider conservation of the amino acid position, pathogenicity predictions and predictions on pre-mRNA splicing. For both individuals, the *LIMK1* variants were considered most likely to cause the phenotype and were studied in patient-derived cells.

#### Bioinformatics

To create Figure 2A, the domain annotations were taken from the entry for the *LIMK1* gene in the NCBI-database (https://www.ncbi.nlm.nih.gov). Figure 2B was created using the sequence alignment as generated by MutationTaster.^57^ Figure 2C was created using programs molscript^58^ and raster3d^59^ and based on the entry pdb 5HVK.^17^ For Figure 2D, the analysis of the conservation of residues in the first LIM domain the NCBI non-redundant protein database was blasted on June 22^nd^ 2022 with the first LIM domains of LIMK1. Sequence hits were filtered for hits with “kinase” in the name. Based on the pairwise alignments with LIMK1 as obtained from the database, the frequency of the amino acid residues that occurs at each position in the LIMK1 sequence was determined and represented in a bar diagram.

#### Glucose measurements

CGM were performed in individual 2 for a period of three consecutive years. In individual 1, glucose was measured for approximately one week. The glucose values were extracted from the Dexcom Clarity Professional portal. Additionally, data from six patients was extracted for comparison. Analysis was performed using R studio (Version 1.4.1106). ‘Non-hypoglycemic days’ were identified as days with all values > 3.5 mmol/L, while ‘Hypoglycemic days’ were quantified as days with one or more glucose values < 3.5 mmol/L. The derivative was calculated by dividing the absolute glucose difference between two time points by the number of minutes between the two time points. Patients could perform additional measurements or turn off their glucose monitor if desired. However, multiple measurements conducted within seconds, or sparse measurements days apart could lead to artificially low- or high glucose variation. As such, we excluded all values where the amount of time (in minutes) between two time points was more or less than 5 min.

#### Assessment of actin polymerization with flow cytometry

Assessment of actin polymerization with Flow Cytometry was performed as described earlier.^60^^,^^61^^,^^62^ Briefly, PBMCs were isolated from the whole blood fraction using Ficoll. Cells were stained with live/dead fixable viability dye (1:1000) and fixed with fix/perm solution (4.2% formaldehyde) and washed with perm/wash buffer (FBS and 0.1% saponin) for 30 min. Afterwards, cells were stained with CD3 (1:50), CD19 (1:100), and phalloidin (1:100) for 1 h at room temperature. Next, cells were analyzed using the LSRFortessa High-Parameter Flow Cytometer. Actin polymerization was assessed by measuring the Median Fluorescence Intensity of the phalloidin staining within CD3 and CD19 cell populations. For fibroblast, we used a slightly different protocol. They were harvested using Accutase and fixed with 4% formaldehyde for 20 min. Afterwards, cells were permeabilized using 0.1% Triton X-100 for 10 min. Fibroblasts were stained with phalloidin (1:100) and DRAQ5 (1:8000). Fibroblasts were analyzed using the LSRFortessa High-Parameter Flow Cytometer. Actin polymerization was assessed by measuring the Median Fluorescence Intensity of the phalloidin staining within fibroblasts.

#### Western blotting

Fibroblasts were harvested using TrypLE and lysed in laemli buffer with protease and phosphatase inhibitors. Immediately after harvesting, samples were boiled for 5 min at 100°. Western blot was performed as described previously.^42^ Membranes were probed with phosphorylated-Cofilin, Cofilin, LIMK1 monoclonal antibody directed against the PDZ domain, or GAPDH antibodies overnight at 4°. INS-1 cells were probed with anti-HA-high-affinity and anti-α-tubulin.

#### Overexpression of *LIMK1* variants in INS-1 cells

Lentiviral plasmids expressing *LIMK1*-WT, or *LIMK1* with variants of individual 1 or 2, were generated by Gibson assembly using gene blocks (Twist Bioscience). All constructs were verified by Sanger sequencing (Quintarabio). Lentiviruses were produced by transfecting HEK293T cells with pLenti-CMV-puro, psPAX2, and pVSVG together with PEI at 1 μg/μL. HEK293T cells were maintained in DMEM with 10% fetal bovine serum under 5% CO_2_ at 37°C. Rat INS-1 cells, originally obtained from the laboratory of Dr. Peter Arvan (University of Michigan), were maintained in RPMI supplemented with 1mM sodium pyruvate, 10% fetal bovine serum, 10mM HEPES and 50 μM β-mercaptoethanol under 5% CO_2_ at 37°C. To generate stable LIMK1 cell lines, INS-1 cells were transduced with lentivirus. 24h after virus transduction, INS-1 cells were selected for ∼48h using 2.5ug/mL puromycin. Transient transfection of INS-1 cells was performed using Lipofectamine 2000 according to the manufacturer’s instructions.

#### Exocytosis measurements in INS-1 cells

INS-1 cells stably expressing hLIMK1 WT or hLIMK1 harboring variants of individual 1 or 2 were plated at a density of 1x10ˆ6 cells in a 6-well plate 72 h prior to the assay in RPMI culturing media supplemented with 10% FBS, 1mM Sodium Pyruvate, and 10mM HEPES. 48 h prior to assay, plated cells were transiently transfected with NPY-pHluorin (a pH-sensitive GFP whose fluorescence is quenched at acidic pH) and NPY-sfCherry3c (a Red Fluorescent Protein that fluoresces regardless of pH). 2 h prior to assay, transfected cells were transferred to glass coverslips. The day of the assay, medium was changed to low Krebs Ringer Bicarbonate (KRB) buffer (1.5mM glucose, 5.4mM KCl). Cells in focus were imaged using a custom built Total internal reflection fluorescence microscopy (TIRF) microscope (Alpha-Plan APO 60×, 1.49 NA) for 15 s under these conditions before the low KRB was replaced with high KRB buffer (40mM Kalium Chloride, 16.7mM Glucose). After changing the medium, images were acquired for another 60 s. Images were acquired every 0.2 s. Exocytotic events were manually counted using the “Multi-Point” tool in ImageJ to indicate the starting frame and coordinates of each event. Event duration was quantified by counting subsequent frames following the initial appearance of each event until it finished.

#### NPY cloning

A gene block of NPY-sfCherry3c was made using the sequence given from Feng et al. (2019).^50^ NPY-sfCherry3c was cloned into pCAGGS backbone (gift from Dr. Edwards Lab, UCSF) using Gibson Assembly, transformed into DH5α cells, and then grown on an Agar plate with Carbenicillin resistance. Colonies were picked and inoculated in Lysogeny Broth overnight for 16 h. Plasmid was purified using “E.Z.N.A. Plasmid DNA Mini Kit I” and sequence was verified via Sanger Sequencing.

#### ELISA methods

hLIMK1 WT, Ala43Thr, or Gly511Ala cells were seeded at 200,000 cells per well in a 24-well plate. The following day, the cells were moved from standard 11.1mM glucose INS-1 media to 5mM glucose INS-1 media for 18 h. Cells were then moved to 1.5mM glucose INS-1 media for 2 h. All cells were then rinsed once with Low KRB buffer. Cells then received 250ul of either Low KRB or High KRB for 2 h. Cells were immediately placed on ice and supernatant containing secreted insulin was collected. Cells were then lysed with 250ul ELISA Lysis buffer (50mM Tris pH8, 300mM NaCl, 1mM EDTA, 2% Triton X-100, 1mM phenylmethylsulfonyl fluoride (PMSF), 1× Protease Inhibitor). Collected supernatants and cellular lysates were then run using “Rat Insulin ELISA Kit” according to manufacturer’s protocol.

#### Phalloidin staining

INS1- cells expressing hLIMK1 WT, Ala43Thr, or Gly511Ala were plated on glass coverslips and then fixed using 4% Paraformaldehyde for 20 min. Cells were treated with Block Buffer (1g Bovine Serum Albumin (BSA), 0.5g Fish Scale gelatin, 10mg Saponin, 50mL Phosphate Buffered Saline) for 1 h and then incubated with Phalloidin-iFluor 488 Reagent at 1:1000 in Block Buffer for 1 h. Coverslips were mounted using DAPI Fluoromount-G and imaged on EVOS-FL-Auto-2 microscope.

Fibroblasts were plated in a 96-well plate with glass bottom at a density of 3.000 cells per well and grown until confluency. The day of the assay, cells were fixed with 4% formaldehyde for 15 min, permeabilized with 0.1% Triton X and stained with phalloidin (1:100) for 1 h at room temperature. Nuclei were counterstained with Hoechst (1:2000) for 5 min. 20X images were shot on the Leica Confocal SP8. 40X images were taken with the EVOS XL.

### Quantification and statistical analysis

Statistical analyses were performed using Prism or R-studio. *p* values ∗*p* < 0.05; ∗∗*p* < 0.01, ∗∗∗*p* < 0.001, ∗∗∗∗*p* < 0.0001 were considered significant. Linear Mixed Models were used for statistical analysis of bar graphs in Figures 3A, 3C, and 3D. One-Way ANOVA followed by Tukey’s multiple comparison test was employed for the analysis of panels in Figures 4A, 4C, 4D, 4G, and 4H). The Robust Regression and Outlier Removal (ROUT) method was used for outlier removal in Figure 4D to analyze the duration of vesicle secretion. Flow cytometry analysis was performed using FlowJo. Exocytosis images were analyzed using ImageJ. Western blots were analyzed using ImageLab software (Version 5.1, BioRad). Figures 1D–1F, 4F, and 4G were created using R and R-studio. The geom_smooth function from the ggplot2 package was employed to generate smoothed trend lines. The following formula was used within geom_smooth: method = “gam”, formula = y ∼ s(x, bs = “cs”, fx = TRUE, k = 60). This formula specifies a generalized additive model (GAM) with a cubic spline basis (bs = 'cs') and other relevant parameters. The phalloidin staining visualized with Confocal was quantified using ImageJ. First, an actin skeleton overlay was created using the FiloQuant package.^63^ Next, the area and aspect ratio of the individual actin bundles were quantified. The number of actin aggregates as part of the total number of actin bundles was calculated by counting all the actin bundles with an area >200 pixels and an aspect ratio <1.5. Since individual 1 has lower levels of actin polymerization, the threshold to detect actin bundles was set slightly lower compared to the other donors.
