# Supplementary material for: LIMK1 variants are associated with divergent endocrinological phenotypes and altered exocytosis dynamics
Source: iScience. 2025 May 5;28(6):112585. doi: 10.1016/j.isci.2025.112585 (PMC12146536; doi:10.1016/j.isci.2025.112585)
Supplement: Document S1. Figures S1–S7 and Tables S1 and S2 [file mmc1.pdf]

iScience, Volume *28*

Supplemental information

LIMK1 variants are associated with divergent endocrinological phenotypes and altered exocytosis dynamics

Irena J.J. Muffels, Theodore Carter, Holger Rehmann, Sebastiaan J. Vastert, Annemarie A. Verrijn Stuart, Andreas C. Blank, Aurore Garde, Bert van der Zwaag, Iris M. De Lange, Jacques C. Giltay, Koen L.I. van Gassen, Klaas Koop, Cedric S. Asensio, and Peter M. van Hasselt

Figure S1: Comparison of pediatric patient controls on a ketogenic diet to individual 1's continuous glucose measurement values, related to Figure 1D/E.

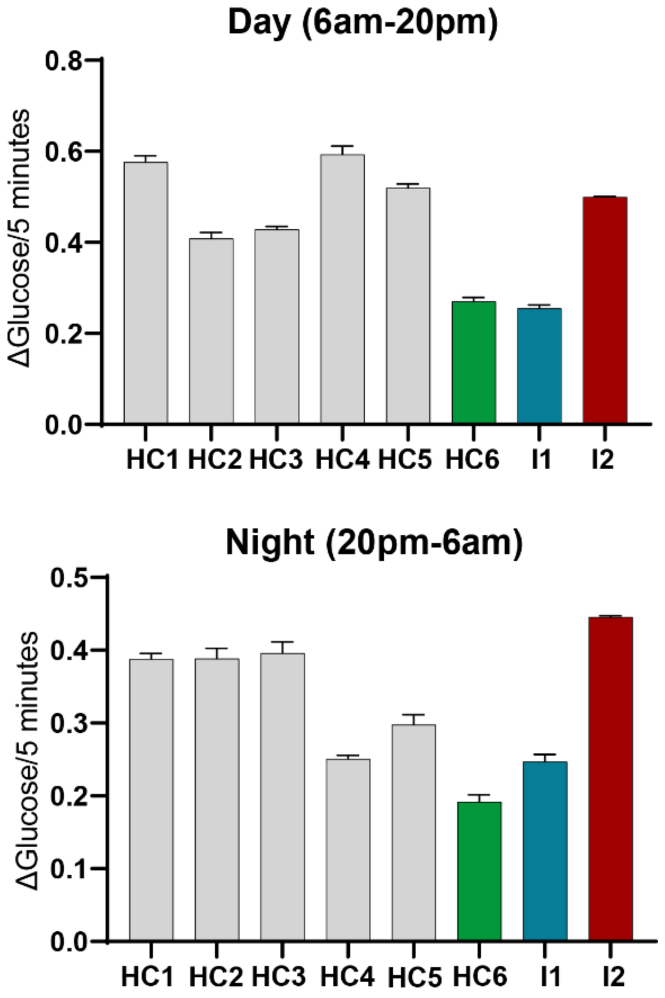

Figure S1: Glucose variation during the day and during the night in 6 pediatric donors and individual 1 and 2 harboring *LIMK1* genetic variants. The bars show the mean glucose variation during the day or during the night for six pediatric controls, individual 1 and individual 2. The green bar represents HC6, who was on a ketogenic diet with limited carbohydrate intake when continuous glucose measurements were initiated. In order to verify whether the glucose variation of individual 2 was similarly the result of low carbohydrate ingestion, the glucose variation between these donors was compared. As can be seen, the low glucose variation is similar to a pediatric control on a ketogenic diet, suggesting that low carbohydrate intake might explain the glucose variation observed.

Figure S2: Additional Western blots, related to Figure 3A/C

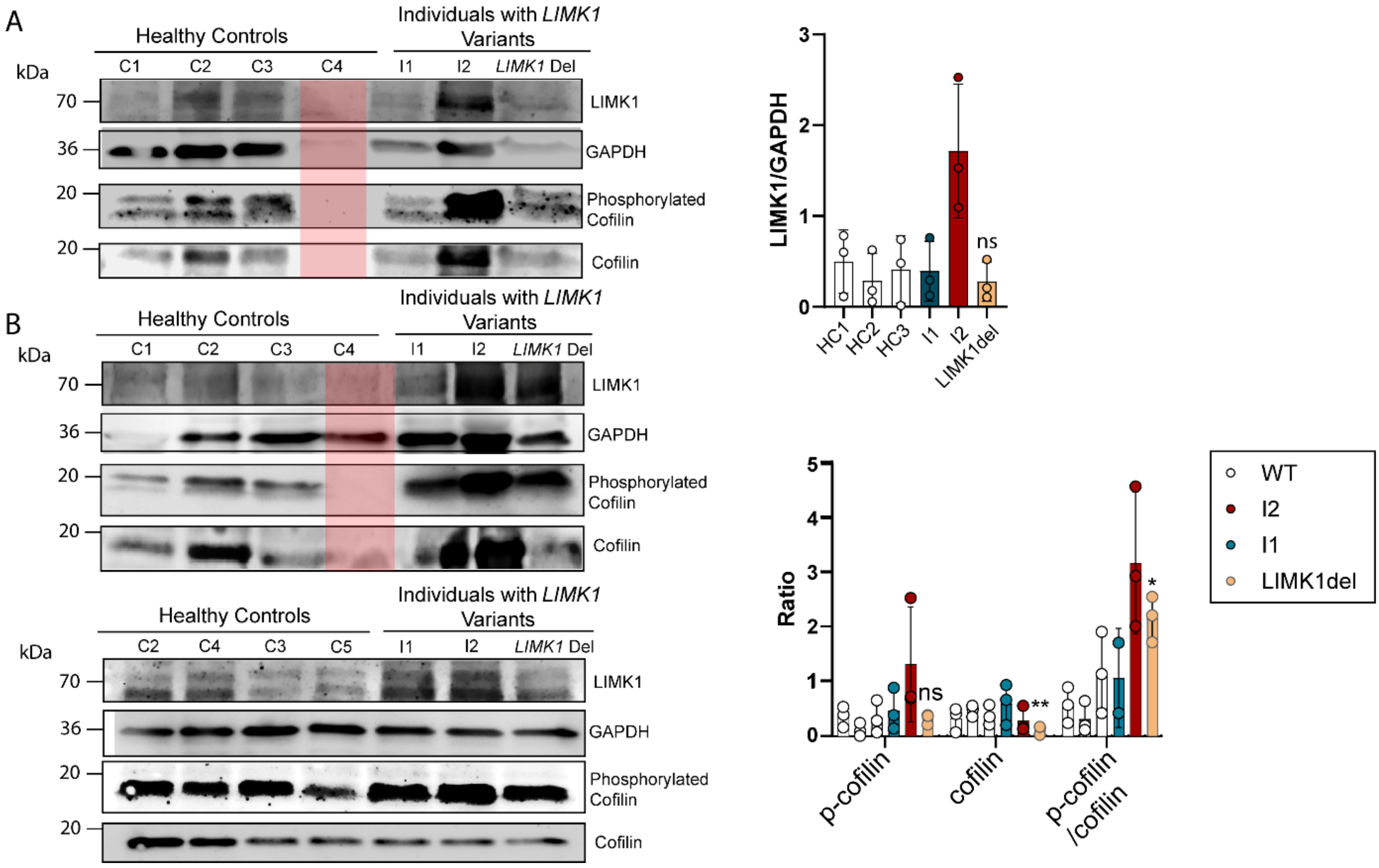

Figure S2: Western blots as referred to in Figure 3A/C
(A) Showing the uncropped version of the western blot which is also shown in Figure 3C in the main body of the manuscript. Healthy control 4 was removed due to the low amount of protein that was present. The bar graphs show the ratio of LIMK1 band intensity normalized to GAPDH. For the bar graph, the three independent western blot experiments show in Figure S2A and B were used. Each bar represents the mean of one patient or healthy control, each dot represents one technical replicate. The bar graphs show the mean values ±SD. Statistics were calculated using linear mixed models. P values *P < 0.05; **P < 0.01, ***P < 0.001, ****P < 0.0001 were considered significant.
(B) Showing the western blots used for quantification incorporated in the bar graphs shown in Figure 3A/C. Again, healthy control 4 was removed from the blot due to low protein levels. GAPDH was used as a housekeeper. The bar graph shows the ratio of cofilin or phosphorylated cofilin over GAPDH. For the bar graph on the right, three independent western blot experiments were used **(Figure S2)**. Each bar represents one biological replicate, each dot represents one technical replicate. The bar graphs show the mean values ±SD. Statistics were calculated using linear mixed models. P values *P < 0.05; **P < 0.01, ***P < 0.001, ****P < 0.0001 were considered significant.

Figure S3: Flow cytometry gating strategy used for B- and T cells, related to Figure 3E

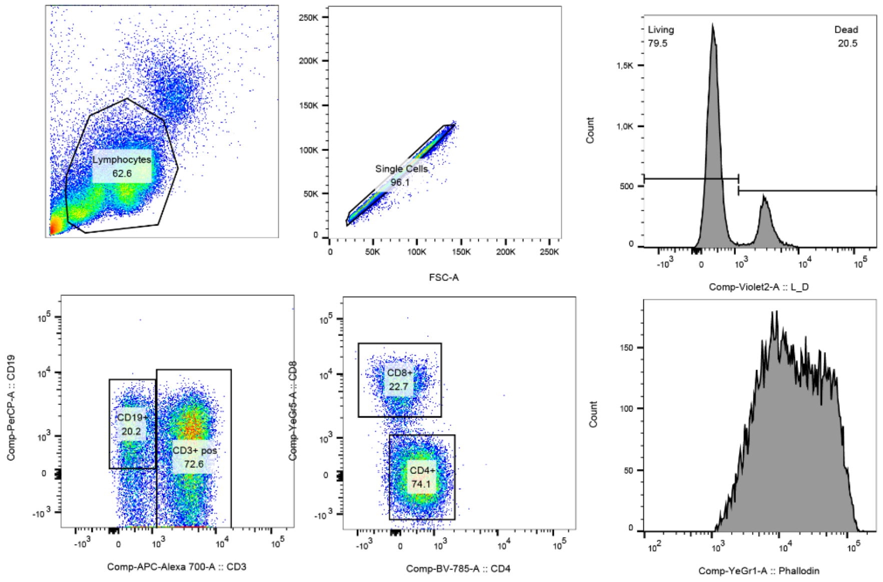

Figure S3: Flow cytometry gating strategy used for B- and T cells
Flow Cytometry Gating Strategy. Lymphocytes were gated using FSC-A and SSC-A. Single cells were gated using SSC-A and SSC-W. Dead cells were excluded from analysis (using a fixable violet dead cell staining). Next, CD3-, CD4- and CD19- cells were gated.

Figure S4: Flow cytometry gating strategy used for fibroblasts, related to Figure 3D

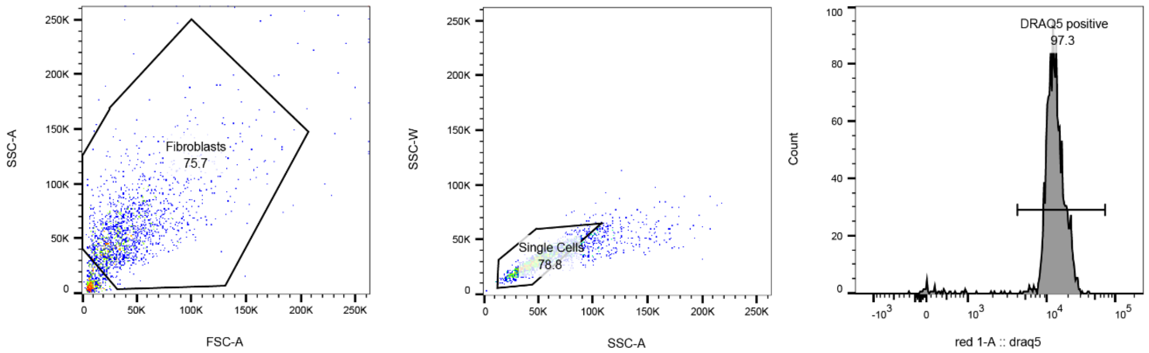

Figure S4: Flow cytometry gating strategy used for fibroblasts
Flow Cytometry Gating Strategy. Fibroblasts were separated from debris using FSC-A and SSC-A. Single cells were gated using SSC-A and SSC-W. Cells without nuclear staining were excluded from analysis.

Figure S5: Phalloidin distribution of healthy controls and individuals with LIMK1 variants, related to Figure 3E

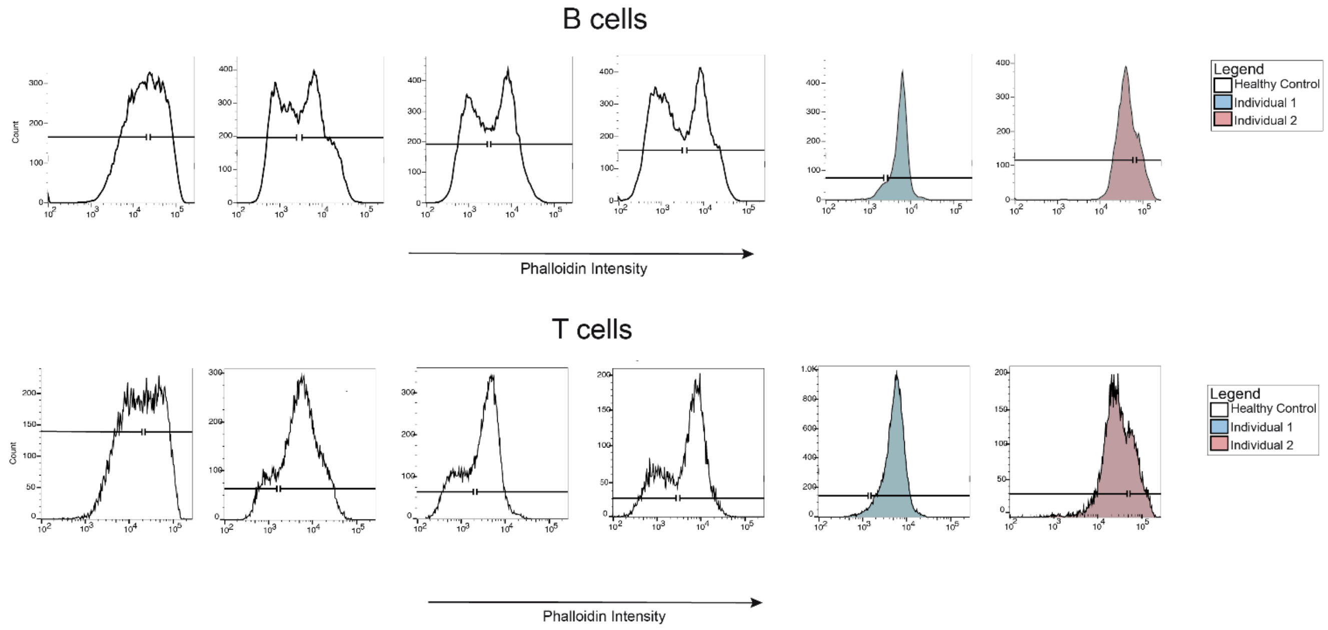

Figure S5: Showing the distribution of the phalloidin staining in four different healthy controls and two individuals with LIMK1 variants in B cells and T cells. The y-axis shows the cellular count, the X-axis shows the intensity of the phalloidin staining.

Figure S6: Phalloidin Staining of fibroblasts derived from individual 1, 2 and the patient with hemizygous LIMK1 deletion, related to Figure 3D/Figure 4A.

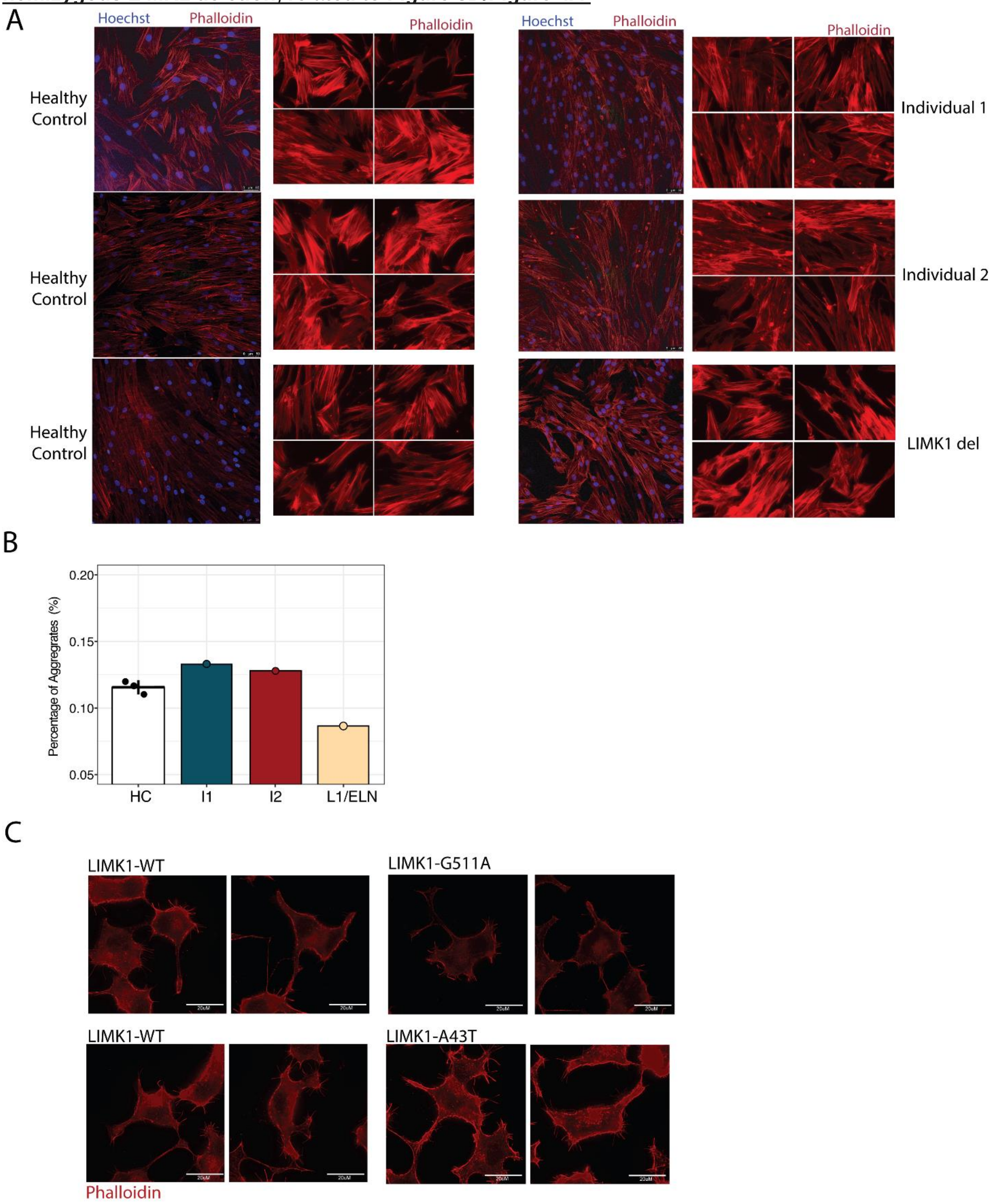

Figure S6: Phalloidin Staining: Showing the results from microscopic evaluation of phalloidin. (A) On the left side, three different healthy controls are shown, while on the right side individuals with *LIMK1* genetic variants are shown. For each patient or healthy control, one representative 20X image is shown, and four representative 40X images are shown. The 20X images were taken with the Leica Confocal SP8, and the 40X images were taken with the EVOS Xl. In individual 1 and 2, a higher number of actin aggregates is observed compared to healthy controls.

(B) Showing the quantification of the percentage of actin aggregates in the 40X images show in Figure S6A. The quantification was performed by calculating the area size and aspect ratio of individual polymerized actin strands and actin aggregates using FiloQuant. All actin strands with an area >200 pixels and Aspect Ratio <1.5 were considered aggregates. The bar graphs show the mean percentage among different donors. The error bars reflect the standard deviation.
(C) INS-1 cells expressing LIMK1-WT, LIMK1-G511A and LIMK1-A43T were stained with phalloidin. On the left side, four different images of INS-1 cells expressing LIMK1-WT are shown. On the right side, two representative images are shown for LIMK1-G511A and LIMK1-A43T expressing INS-1 cells.

Figure S7: Western blot of HA-tagged LIMK1 in INS-1 cells, related to Figure 4

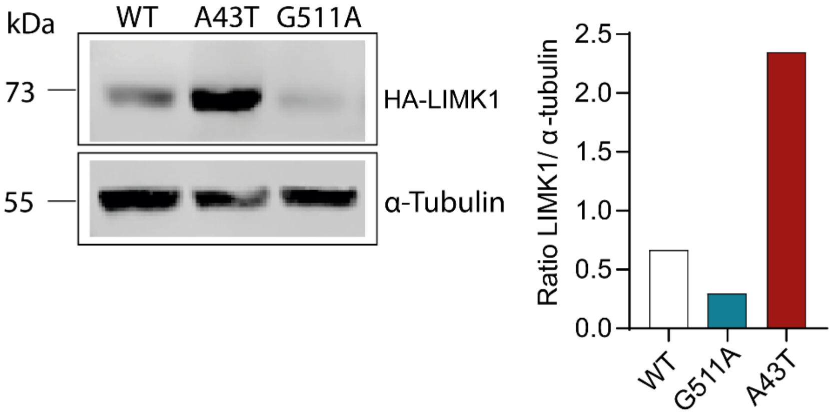

Figure S7: Western blot results.
Western blot of INS-1 cells overexpressing HA-WT-LIMK1, or HA-LIMK1 harboring variants of individual 1 and 2. The western blot shows increased LIMK1 protein levels for the INS-1 cells expressing LIMK1-Ala43Thr, while INS-1 cells overexpressing LIMK-Gly511Ala seem to have decreased LIMK1 protein levels compared to overexpression of WT-LIMK1.

Table S1: Genetic variants identified with Whole Exome Sequencing in Individual 1 and 2

**Individual 1**						
**Gene**	**Transcript**	**Inheritance**	**cDNA**	**Protein**	**Mutation Taster**	**Reason for In-/ Exclusion**
SLC3A1	NM_000341.3	Heterozgous; AD; Mother has the same variant	c.1400T>C	p.Met467Thr	Known variant; risk factor for cytinuria (rs18115)	Cystinuria (OMIM 220100) does not corelate with patient phenotype, mother is carrier of the same variant.
ACAA1	NM_001607.3	Heterogyzous De Novo	c.514G>A	p.Glu172Lys	Polymorphism	Predicted as polymorphism, amino acid position not conserved (BP4)
LIMK1	NM_002314.3	Heterozgyous De Novo	c.1532G>C	p.Gly511Ala	Disease causing	Predicted as disease causing, amino acid position highly conserved, absent from population databases.
CEP78	NM_00134 98 39.1	Heterozygous De Novo	c.254-7G>A	N/A	N/A	Not predicted to affect pre-mRNA splicing (BP4)
NFRKB	NM_00114 38 35.1	Heterozygous De Novo	c.1755C>T	p.Asp585=	N/A	Not predicted to affect pre-mRNA splicing (BP4)
BTBD7	NM_00100 28	Heterozgyous	c.2122-13delT	N/A	N/A	Small deletions in intronic T repeat. Not

	60.3	De Novo				predicted to affect pre-mRNA splicing (BP3)
MUC16	NM_024690.2	Heterozgyous De Novo	c.40674_40677 delAAGC insAAGCTGGT	p.Pro13560Trpfs*22	N/A	Dismissed based on quality parameters (low coverage, strand bias): artefact.
**Individual 2**						
LIMK1	NM_002314.3	Heterozgyous De Novo	c.127G>A	p.Ala43Thr	Polymorphism	Followed up because it was the only potential candidate that remained after filtering.

Table S1: Genetic variants found after filtering using Trio Whole Exome Sequencing in both individuals. MAF = Minor Allele Frequency. Green colored rows indicate that the variant identified through Whole Exome Sequencing met one or more of the criteria of the ACMG to be benign, and the variant was considered less likely to cause the phenotype of the patient. The column on the far right indicates the reason for exclusion of the variant. If the row is colored red, the variant did not meet any of the criteria of the ACMG to be benign. For individual 2, the LIMK1 variant was the only variant that remained after filtering and was therefore followed up, despite prediction algorithms predicting it to be benign.

Table S2: Detailed information about diet, diagnosis and glucose measurements of pediatric controls and individuals with *LIMK1* variants, related to Figure 1E.

Donor Name	No. of days with continuous glucose measurement	Diagnosis	Diet	Hypoglycemic Days
Individual 2	>900	No Diagnosis	Hypercaloric diet, nightly feeding	Yes
Individual 1	8	No Diagnosis	Ketogenic Diet	No
HC1	18	GSD type I	Continuous nightly feeding	Yes
HC2	9	Pediatric hypoglycemia	Normal Diet	Yes
HC3	8	Hypoglycemia E.C.I.	Normal Diet	No
HC4	23	GSD type I	Continuous nightly feeding	Yes
HC5	8	Fasting Intolerance E.C.I.	Normal Diet	No
HC6	8	No Diagnosis	Ketogenic Diet	Yes

Table S2: Detailed information about diet, diagnosis and glucose measurements of pediatric controls and individuals with *LIMK1* variants.
Detailed information about the pediatric controls used as reference and individuals with *LIMK1* genetic variants used in Figure 1C. E.C.I. refers to e causa ignota, meaning the cause was unknown.
